# Mapping the Seattle Angina Questionnaire to EQ-5D-5L in patients with coronary heart disease

**DOI:** 10.1186/s12955-023-02151-9

**Published:** 2023-07-03

**Authors:** Chaofan Li, Lei Dou, Qiang Fu, Shunping Li

**Affiliations:** 1 Centre for Health Management and Policy Research, School of Public Health, Cheeloo College of Medicine, Shandong University, Wenhua Xi Road 44, Jinan, 250012 China; 2 NHC Key Lab of Health Economics and Policy Research, (Shandong University), Wenhua Xi Road 44, Jinan, 250012 China; 3 Center for Health Preference Research, Shandong University, Wenhua Xi Road 44, Jinan, 250012 China; 4 Department of Cardiovascular Surgery, General Hospital of Tianjin Medical University, Anshan Road 154, Tianjin, 300051 China

**Keywords:** Coronary heart disease, Health-related quality of life, Seattle angina questionnaire, Health utility, EQ-5D-5L

## Abstract

**Background:**

Health economic evaluation is critical in supporting novel cardiovascular disease therapies. However, most clinical studies do not include preference-based questionnaires to calculate utilities for health economic evaluations. Thus, this study aimed to develop mapping algorithms that convert the Seattle Angina Questionnaire (SAQ) to EQ-5D-5L health utility scores for patients with coronary health disease (CHD) in China.

**Methods:**

Data were obtained from a longitudinal study of patients with CHD conducted at the Tianjin Medical University General Hospital in China. Convenience sampling was used to recruit patients with CHD. The inclusion criteria were having been diagnosed with CHD through a medical examination and being aged 18 years or older. The exclusion criteria were a lack of comprehension ability, serious comorbidities, mental illness, and hearing or vision impairment. All eligible patients were invited to participate, and 305 and 75 patients participated at baseline and in the follow-up, respectively. Seven regression models were developed using a direct approach. Furthermore, we predicted the five EQ-5D items using ordered logit model and derived the utility score from predicted responses using an indirect approach. Model performances were evaluated using mean absolute error (MAE), root mean squared error (RMSE), correlation coefficient (ρ), and Lin’s concordance correlation coefficient (CCC). A five-fold cross-validation method was used to evaluate internal validation.

**Results:**

The average age was 63.04 years, and 53.72% of the included patients were male. Most (70.05%) patients had unstable angina pectoris, and the mean illness duration was 2.50 years. The EQ-5D scores were highly correlated with five subscales of the SAQ, with Spearman’s rank correlation coefficients ranging from 0.6184 to 0.7093. The mixture beta model outperformed the other regression models in the direct approach, with the lowest MAE and RMSE and highest ρ and CCC. The ordered logit model in the indirect approach performed the same as the mixture beta regression with equal MAE, lower RMSE, and higher ρ and CCC.

**Conclusion:**

Mapping algorithms developed using mixture beta and ordered logit models accurately converted SAQ scores to EQ-5D-5L health utility values, which could support health economic evaluations related to coronary heart disease.

**Supplementary Information:**

The online version contains supplementary material available at 10.1186/s12955-023-02151-9.

## Background

Cardiovascular disease (CVD) is the leading cause of premature deaths across the world [1]. It is estimated that approximately 17.9 million patients die from CVDs globally, representing 32% of all deaths [2]. Coronary heart disease (CHD) is a major category of CVD, accounting for one-third of all deaths in adults older than 35 years [3]. Internationally, China is the largest developing country and has the highest burden of CHD, with a mortality rate of 115.32 per 100,000 in urban and 122.04 in rural areas in 2017 [4]. It is estimated that CHD treatment account for 7% of the total health expenditure in Beijing, China [5]. Furthermore, the morbidity and hospitalisation rates of CHD are rapidly increasing in China [4, 6]. To cope with the serious challenges of CHD epidemics, health systems must develop cost-effective strategies to allocate healthcare resources and control disease burden [7].

Health economic evaluations, such as cost-utility analysis (CUA), are widely used to decide on healthcare resource allocation and health insurance reimbursement mechanisms [8]. The measurement and validation of health utility value are critical for performing CUA [9]. Generic preference-based questionnaires, such as EQ-5D and SF-6D, are commonly used to estimate health utility value [10, 11]. The EQ-5D is a reliable and valid tool to measure health utility values in patients with CVD [11]. However, most clinical studies do not include preference-based questionnaires, although they usually include disease-specific questionnaires [12]. When a preference-based measure is not available in a clinical study, “mapping” could estimate health utility values from non-preference disease-specific measures to generic preference-based instruments using statistical association [12]. Recently, mapping has been increasingly used to estimate health utility values for conducting health economic evaluations and CUA [10, 13].

The disease-specific Seattle Angina Questionnaire (SAQ) is widely used to evaluate patients with CHD [14–16]. To estimate the health utility values of CHD, previous studies have developed several mapping algorithms using direct approaches [17, 18]. However, most of these studies were conducted prior to 2010 [19]. Furthermore, previous studies have only used the direct mapping approach in western countries, in which the algorithms cannot estimate health utility by applying other countries’ data. Research on mapping algorithms for patients with CHD in Asia is scarce [13]. Therefore, this study aimed to develop mapping algorithms to predict EQ-5D values based on SAQ scores using direct and indirect approaches among patients with CHD.

## Methods

### Data

We obtained data from a longitudinal study of patients with CHD conducted at the Tianjin Medical University General Hospital, China. Convenience sampling was used to recruit patients with CHD. The inclusion criteria were having been diagnosed with CHD through a medical examination and being aged 18 years or older. The exclusion criteria were a lack of comprehension ability, serious comorbidities, mental illness, and hearing or vision impairment. Details of sample size calculation and ethics approval are provided elsewhere [20]. The baseline survey was conducted in-person from April to September 2019 by trained interviewers, and follow-up interviews were conducted four weeks after discharge by phone. All eligible patients were invited to participate, and 305 and 75 patients participated at baseline and in the follow-up, respectively.

### Measurement

EQ-5D-5L is a widely used preference-based questionnaire with five dimensions to measure health utility: mobility, self-care, usual activities, pain/discomfort, and anxiety/depression [21]. Items are scored on a five-point scale (1 = no problem; 5 = extreme problem). The Chinese version of EQ-5D-5L was used to calculate health utility scores, which range from − 0.391 to 1 [22]. A value of 1 represents full health, 0 represents death, and negative values represent health states considered worse than death.

The SAQ, a widely used disease-specific instrument for adult patients with CHD, was used to assess HRQoL in this study [23]. The questionnaire comprises 19 items categorized into five scales:1) physical limitation (PL), 2) anginal stability (AS), 3) anginal frequency (AF), 4) treatment satisfaction (TS), and 5) disease perception (DP). Each item is assessed using an ordinal value ranging from 1 to 5 or 6, with higher item scores representing a high level of function or satisfaction. Five subscale scores were calculated separately and no total scores were generated: first, summing item scores within each subscale; second, transforming subscale scores to a 0–100 by subtracting possible lowest values, dividing by the range of the subscale, and multiplying by 100 [23]. A higher subscale score represents fewer functional limitations or is more satisfied [18]. In addition, the participants’ age, gender, disease type (stable angina pectoris, unstable angina pectoris, and myocardial infarction), and illness duration were obtained.

### Statistical analysis

#### Descriptive statistics

The participants’ characteristics were described using summary statistics, with mean and standard deviation (SD) for continuous variables and frequency and percentage for categorical variables. The Shapiro–Wilk test was used to evaluate the normality of continuous variables, and a histogram was drawn to visually display the distribution of EQ-5D-5L values. Spearman’s rank correlation coefficient was used to assess the conceptual overlap between EQ-5D-5L and the five subscales of the SAQ. Following a previous study [24], we pooled the baseline and follow-up data to develop the mapping algorithms.

#### Model development

Following the recommendations of best practice [12, 25, 26], both direct and indirect approaches were used to develop the mapping algorithms. Direct mapping approaches directly predict utility value using ordinary least squares (OLS) and other regression models based on SAQ. Following Gray [27], we used indirect mapping. First, we estimated response probabilities for EQ-5D-5L items using the multinomial logit regression model. Second, we calculated utility value based on response probability and EQ-5D-5L value.

Direct mapping has several regression techniques, and OLS is the most common method to map EQ-5D [10]. Following previous studies, we first used OLS to estimate the linear relationship between EQ-5D-5L and SAQ. However, the OLS was not considered be appropriate if the outcome variable data violate the assumption of normal distribution or homogeneous variance [28]. Then, generalized linear model (GLM) was used to fit the EQ-5D-5L values with a skewed distribution [29]. In this GLM model, log was chosen as the link function, and Gaussian was set as the distribution family. Given that the EQ-5D-5L score is censored and inflated at 1, both OLS and GLM could produce a system bias and inefficiency estimation [30]. Thus, we used a Tobit and censored least absolute deviation (CLAD) regression model to deal with right-censoring at a score of 1 in EQ-5D-5L [26]. Moreover, to address the potential bias of outliers and heteroskedasticity, a robust MM estimator (RMM) was fitted in this study. Chen et al. introduced RMM into the mapping approach; it has been shown to have both high breakdown points and efficiency [31]. A previous study proved that bespoke mixture models, including the adjusted limited dependent variable mixture model (ALDVMM) and the mixture beta regression model (BM), had better performance than traditional regression models [32]. The ALDVMM is an econometric model developed to fit variables having an upper bound at1, a large gap between 1 and the next set of feasible values, and multimodal distribution [33]. The BM is a two-part model to fit skewed EQ-5D-5L scores that are unimodal or multimodal [32]. The first part is a beta mixture model to fit utility scores between 0 and 1, and the second part is a multinomial logit model to fit the masses of boundary values (e.g., full health) [34]. The ALDVMM and BM can be easily implemented using the Stata command “aldvmm” and “betamix”, respectively [34, 35].

For the indirect approach, the ordered logit model was used to examine the relationship between EQ-5D-5L values and SAQ scores. Subsequently, we predicted probabilities for EQ-5D-5L dimensions [36] and calculated EQ-5D-5L scores.

#### Model performance

Model performance was evaluated using four indicators based on prediction: mean absolute error (MAE), root mean squared error (RMSE), Pearson correlation coefficient (ρ), and Lin’s concordance correlation coefficient (CCC) [37]. The MAE is the mean value of the absolute differences between the observed and predicted EQ-5D-5L scores, and RMSE is the root of the expected value of the squared difference between the observed and predicted scores [38]. The ρ and CCC quantify the agreements between observed and predicted values. CCC is robust for evaluating predictive performance against data from uniform and other distributions [39]. It was noted that the model performed better, with lower MAE and RMSE and higher correlation coefficients. The MAE is considered the primary criterion to evaluate model performance, as it is a natural measure of average differences between the observed and predicted values, unambiguous, and robust to outliers [40, 41]. Based on the best-performing model, the final mapping algorithm was estimated using the full sample. Furthermore, we drew scatter and line plots of the observed values versus the predictions to visually model the performance. Furthermore, we calculated the observed and predicted changes in EQ-5D-5L scores of 75 patients with CHD between the baseline and follow-up surveys. Moreover, we conducted data analysis and mapping using baseline data and presented the results in Supplementary Material 1.

No suitable external dataset was available for validating the model predictions. Thus, following previous studies, we used a 5-fold cross-validation method to validate the model [28, 30, 42]. First, the participants were randomly divided into five subgroups. Second, four subgroups (80%) were used as training samples to develop the mapping algorithm, and the remaining subgroup (20%) was used as validation samples to evaluate the predictive performance. Third, this process was repeated five times so that all subgroups could be used as both training and validation samples.

All statistical analyses were performed using Stata 15.0 [43].

## Results

### Basic characteristics

Table 1 displays the participant characteristics. The mean (SD) age was 63.04 (9.68) years, and the proportion of male participants (53.72%) was slightly higher than that of female (46.28%). The majority of patients (70.05%) had unstable angina pectoris, while 21.70% and 8.24% had unstable angina pectoris and myocardial infarction, respectively. The average duration of illness of the participants was 2.50 years. Participant characteristics from the baseline and followed-up surveys are presented in Supplementary Material 1.

**Table 1:** Descriptive statistics of the observations

Variables	
Age (years), Mean (SD)	63.04 (9.68)
Gender	
Male, *N* (%)	202 (53.72)
Female, *N* (%)	174 (46.28)
Disease type	
Stable angina pectoris, *N* (%)	79 (21.70)
Unstable angina pectoris, *N* (%)	255 (70.05)
Myocardial infarction, *N* (%)	30 (8.24)
Duration of illness (years), Mean (SD)	2.50 (5.54)
EQ-5D-5L, Mean (SD)	0.87 (0.14)
Seattle angina questionnaire	
Physical limitation, Mean (SD)	70.83 (14.41)
Anginal stability, Mean (SD)	36.18 (34.07)
Angina frequency, Mean (SD)	64.55 (28.06)
Treatment satisfaction, Mean (SD)	67.69 (11.62)
Disease perception, Mean (SD)	51.80 (13.77)

**Fig. 1:**
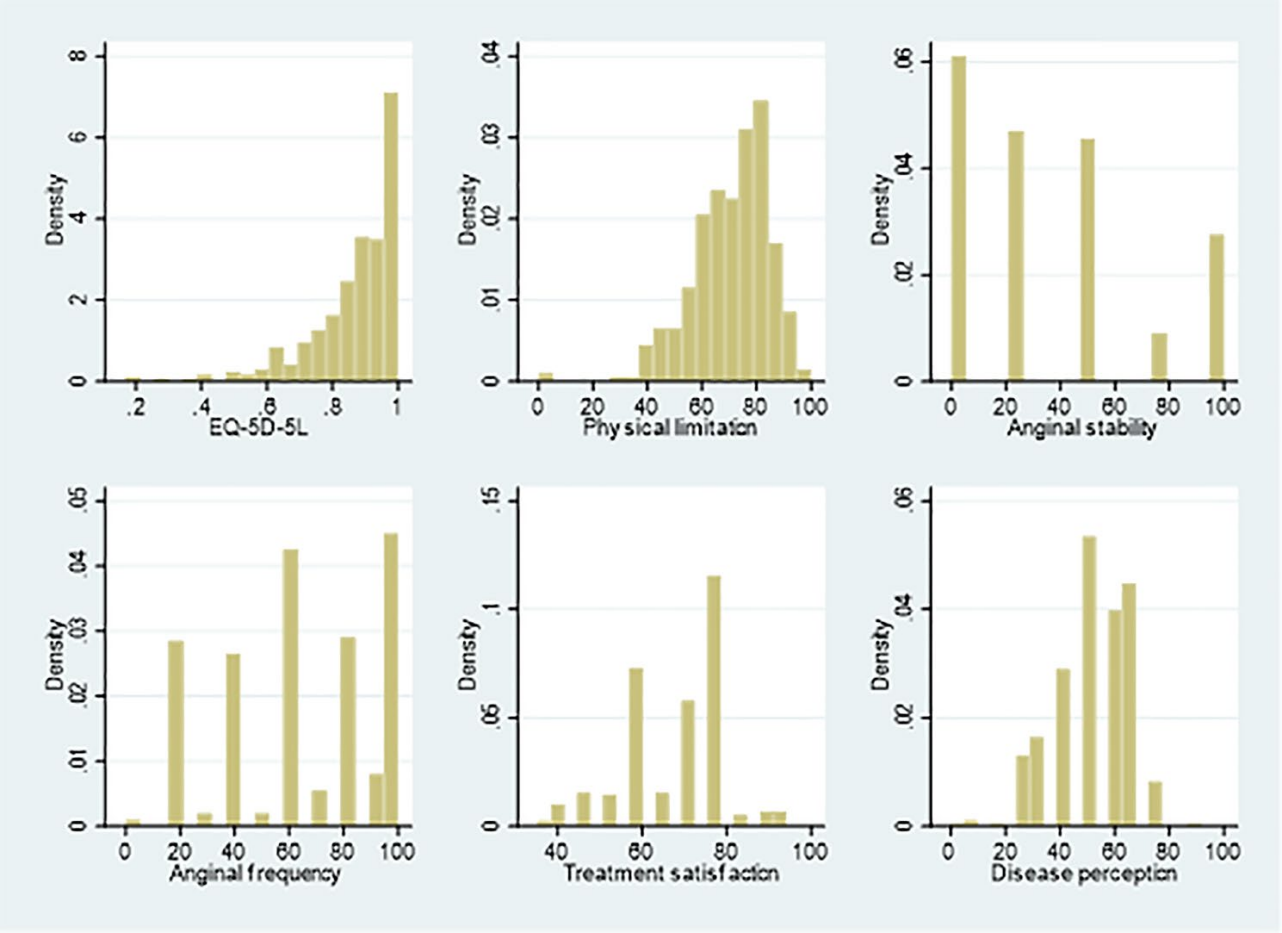
Distribution of EQ-5D-5L and five subscales of Seattle angina questionnaire

The mean (SD) of EQ-5D-5L score was 0.87 (0.14). As Fig. 1 shows, EQ-5D-5L ranged from 0.17 to 1, with left skewness and a high proportion (31.1%) of full health. As for the five subscales of the SAQ, anginal stability had the lowest mean (36.18) and highest standard deviation (34.07), whereas the other four subscales had higher means (ranging from 51.80 to 70.83) and lower standard deviation (from 11.62 to 14.41). Moreover, the subscales of the SAQ also had a skewed distribution. Further Shapiro-Wilk tests showed that both EQ-5D-5L scores and subscales of the SAQ were non-normally distributed (*P* < 0.001).

### EQ-5D-5L utility score prediction and goodness-of-fit

As Table 2 shows, there was a high positive correlation between EQ-5D-5L scores and the five subscales of the SAQ, ranging from 0.6184 to 0.7093 and significant at 0.01 level. Moreover, there were moderate or high significant negative correlations between the five subscales of the SAQ and the four dimensions of EQ-5D, including mobility, usual activity, pain/discomfort, and anxiety/depression. However, there was a significantly low correlation between the SAQ subscales and self-care, in which the absolute values of Spearman correlation coefficients were smaller than 0.3.

**Table 2:** Spearman’s correlation coefficients of EQ-5D-5L and Seattle Angina Questionnaire subscales

	EQ-5D-5L	MO	SC	UA	PD	AD
SAQ PL	0.6416^**^	-0.5907^**^	-0.2598^**^	-0.5751^**^	-0.5107^**^	-0.3838^**^
SAQ AS	0.6502^**^	-0.4444^**^	-0.1958^**^	-0.4806^**^	-0.5855^**^	-0.4770^**^
SAQ AF	0.6676^**^	-0.4647^**^	-0.2203^**^	-0.4988^**^	-0.5865^**^	-0.4899^**^
SAQ TS	0.6184^**^	-0.4128^**^	-0.1731^**^	-0.4507^**^	-0.5807^**^	-0.4591^**^
SAQ DP	0.7093^**^	-0.3765^**^	-0.1170^*^	-0.4661^**^	-0.6353^**^	-0.6384^**^

^*^, *P* < 0.05; ^**^, *P* < 0.01

*Notes*: MO, mobility; SC, self-care; US, usual activities; PD, pain/discomfort, AD, anxiety/depression. PL, physical limitation; AS, angina stability; AF, angina frequency; TS, treatment satisfaction; DP, disease perception

**Fig. 2:**
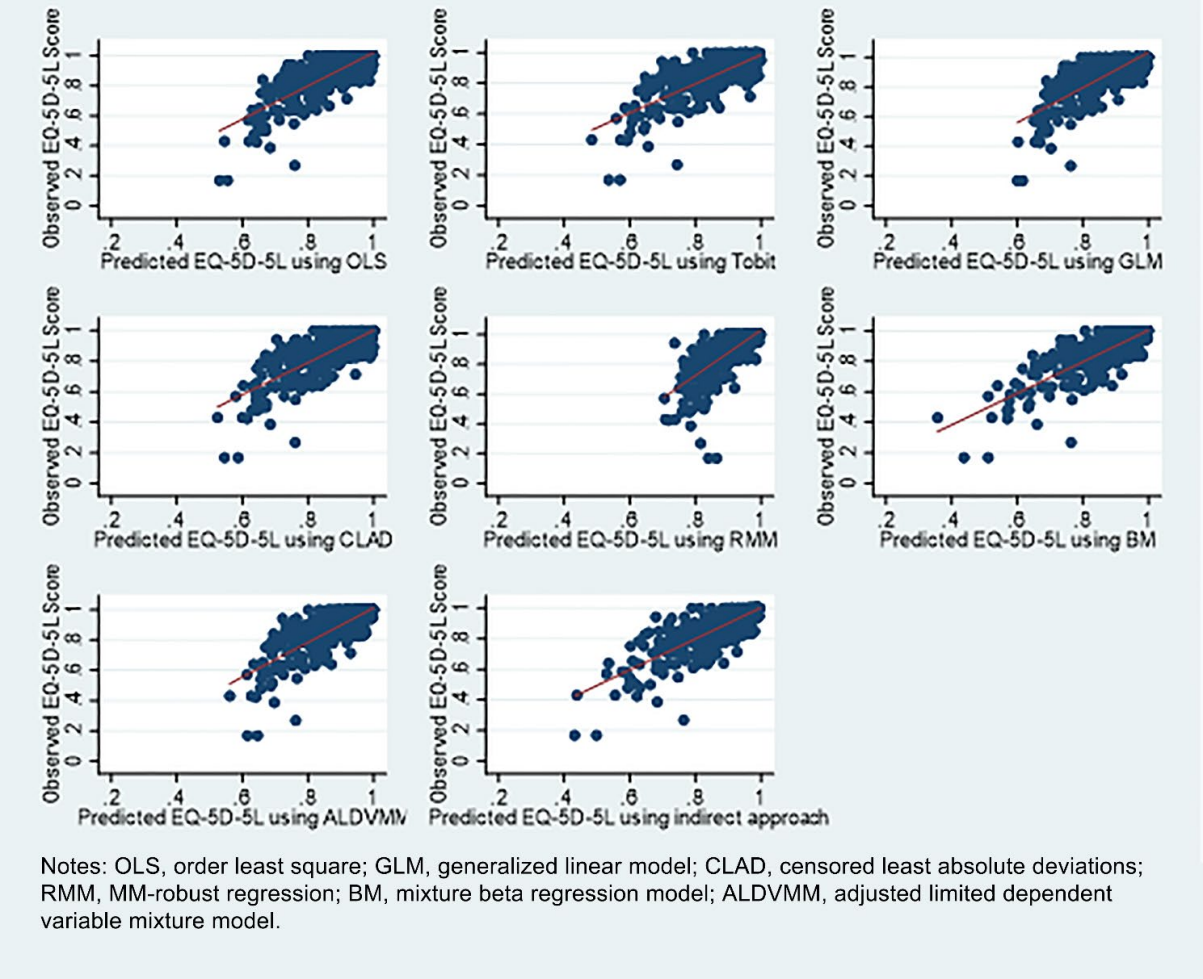
Scatter plot of observed and predicted EQ-5D-5L utility scores. *Note*s: OLS, order least square; GLM, generalized linear model; CLAD, censored least absolute deviations; RMM, MM-robust regression; BM, mixture beta regression model; ALDVMM, adjusted limited dependent variable mixture model

Table 3 displays the predicted scores and goodness-of-fit for the entire sample. As shown in Supplementary Material 2, age, sex, disease type, and illness duration were found to be non-significant in most of the regression models; thus, they were not included in the equations. The mean of the predictive EQ-5D-5L scores obtained using OLS regression (0.8725) was equivalent to the observed mean. For the 75 followed patients, the mean of the observed change in EQ-5D-5L scores between the baseline and follow-up surveys was 0.1256, with the closest predicted value of 0.1304 captured from OLS regression. The minimum estimated utility score ranged from 0.2985 to 0.7057, all of which were higher than those observed. Contrastingly, the maximum predicted utility score captured from most of the models was larger than 1, except for BM, ALDVMM, and the indirect approach. Figure 2 also displays similar results; that is, most of the models had overpredictions for patients with poor health and underpredictions for those with good health. The BM and ordered logit models could predict EQ-5D-5L scores under 0.4, while other models usually overestimated the values.

**Table 3:** Goodness of fit of direct and indirect mapping approach from full sample

Model	Mean	Minimum	Maximum	Mean changes between baseline and follow-up survey	MAE	RMSE	ρ	CCC
Observed	0.8725	0.1690	1.0000	0.1256	-	-	-	-
OLS	0.8725	0.5302	1.0877	0.1304	0.0656	0.0889	0.7846	0.7621
Tobit	0.9090	0.4843	1.2055	0.1990	0.0843	0.1058	0.7788	0.7539
GLM	0.8729	0.6004	1.0980	0.1313	0.0678	0.0923	0.7653	0.7331
CLAD	0.8781	0.6384	1.0730	0.1444	0.0646	0.0960	0.7469	0.6910
RMM	0.9008	0.7057	1.0555	0.0993	0.0653	0.1066	0.7284	0.5797
BM	0.8734	0.3573	0.9990	0.1160	**0.0566**	0.0813	**0.8267**	**0.8063**
ALDVMM	0.8776	0.5616	0.9991	0.1130	0.0592	0.0877	0.8010	0.7529
Indirect approach	0.8663	0.2985	0.9961	0.1176	0.0572	0.0796	**0.8333**	**0.8243**

*Notes*: Bold number indicate a best result on that indicator. OLS, order least square; GLM, generalized linear model; CLAD, censored least absolute deviations; RMM, MM-robust regression; BM, mixture beta regression model; ALDVMM, adjusted limited dependent variable mixture model. ρ, Pearson’s correlation coefficients between observed EQ-5D-5L scores and prediction; MAE, mean absolute error; RMSE, root mean square error, CCC, Lin’s Concordance correlation coefficient

### Validation and prediction performance

Table 4 reports the four performance indicators using cross validation method. In the direct mapping models, BM was chosen four times as the most accurate prediction model, which showed the lowest value of MAE (0.0591) and RMSE (0.0864) and highest value of ρ (0.8022) and CCC (0.7819). The ALDVMM was a sub-optimal model based on ρ (0.7826), MAE (0.0612) and RMSE (0.7396), whereas its CCC was lower than that of BM (0.7819), Tobit (0.7539) and OLS (0.7447). As for indirect mapping approach, ordered logit model had comparable or better predictive performance than the models in direct approach. The MAE of ordered logit model was 0.0591, equivalent to that of BM. Moreover, its value of RMSE (0.0840) was lower than that of BM (0.0864), and ρ and CCC were 0.8113 and 0.7903 which were higher than those of BM (0.8022 and 0.7819, respectively).

**Table 4:** Predictive performance of direct and indirect mapping approach using cross validation method

Model	MAE	RMSE	ρ	CCC
OLS	0.0673	0.0919	0.7683	0.7447
Tobit	0.0865	0.1095	0.7628	0.7539
GLM	0.0691	0.0948	0.7507	0.7182
CLAD	0.0671	0.1089	0.7660	0.7036
RMM	0.0666	0.1080	0.7153	0.5691
BM	**0.0591**	**0.0864**	**0. 8022**	**0.7819**
ALDVMM	0.0612	0.0907	0.7826	0.7396
Indirect approach	**0.0591**	**0.0840**	**0.8113**	**0.7903**

*Notes*: Bold number indicate a best result on that indicator. OLS, order least square; GLM, generalized linear model; CLAD, censored least absolute deviations; RMM, MM-robust regression; BM, mixture beta regression model; ALDVMM, adjusted limited dependent variable mixture model. ρ, Pearson’s correlation coefficients between observed EQ-5D-5L scores and prediction; MAE, mean absolute error; RMSE, root mean square error, CCC, Lin’s Concordance correlation coefficient

### Mapping equations

Table 5 presents the coefficients for predicting EQ-5D-5L score from SAQ subscales. Variance-covariance matrixes are presented in Supplementary Material 3. As for direct approach, physical limitation and disease perception were robustly significant in all equations, and anginal stability and anginal frequency were significant in most of the regression models. However, treatment satisfaction was not significant in most of the models except for in CLAD.

**Table 5:** Regression coefficients for predicting EQ-5D-5L health utility scores from Seattle Angina Questionnaire using direct approach, *N* = 380

Variable	OLS	Tobit	GLM	CLAD	RMM	BM		ALDVMM	
						C1_mu	PM_ub	Com 1	Com 2
SAQ PL	0.5271^***^	0.6040^***^	0.5712^***^	0.2603^***^	0.1500^***^	3.7922^***^	5.3271^**^	0.0834	0.8770^***^
	[0.0371]	[0.0471]	[0.0462]	[0.0329]	[0.0363]	[0.3346]	[1.6583]	[0.0548]	[0.0730]
SAQ AS	0.0381	0.0934	0.0431	0.0276	0.0303^*^	0.4226	1.4955^*^	0.0529	0.0821
	[0.0213]	[0.0299]	[0.0244]	[0.0207]	[0.0135]	[0.2691]	[0.7072]	[0.0281]	[0.0606]
SAQ AF	0.0538^*^	0.0838^*^	0.0634^*^	0.0983^***^	0.0674^*^	0.6138^**^	1.9211	0.0557	0.0973
	[0.0255]	[0.0323]	[0.0313]	[0.0249]	[0.0276]	[0.2241]	[1.0112]	[0.0360]	[0.0582]
SAQ TS	0.1018	0.1211	0.0939	0.1737^*^	0.0214	0.6172	1.9462	0.0087	0.2256
	[0.0602]	[0.0767]	[0.0728]	[0.0573]	[0.0629]	[0.5729]	[2.5628]	[0.0753]	[0.1383]
SAQ DP	0.1491^**^	0.2431^**^	0.1890^**^	0.1343^***^	0.2725^***^	1.3959^**^	10.2317^***^	0.3462^***^	0.0341
	[0.0529]	[0.0680]	[0.0650]	[0.0471]	[0.0498]	[0.4715]	[2.4454]	[0.0621]	[0.1164]
Constant	0.3046^***^	0.1855^***^	-0.7661^***^	0.4421^***^	0.5844^***^	-1.5982^***^	-13.9807^***^	0.6063^***^	-0.0005
	[0.0345]	[0.0452]	[0.0425]	[0.0317]	[0.0388]	[0.3250]	[2.0050]	[0.0617]	[0.0801]

*Notes*: ^*^, *P* < 0.05; ^**^, *P* < 0.01; ^***^, *P* < 0.001; Com, component; C1_mu, Component 1 of mixture model; PM_ub, the inflation part of the model at perfect health

OLS, order least square; GLM, generalized linear model; CLAD, censored least absolute deviations; RMM, MM-robust regression; BM, mixture beta regression model; ALDVMM, adjusted limited dependent variable mixture model. The coefficients were captured from each regression model in which Seattle Angina Questionnaire score multiplied by 100

Regarding the coefficients of indirect mapping displayed in Table 6, physical limitation was found to be a robustly significant negative predictor for the four dimensions of the EQ-5D, except for anxiety/depression. Anginal stability was significant in predicting pain/discomfort, while anginal frequency was significant in estimating usual ability, pain/discomfort, and anxiety/depression. Treatment satisfaction was a significant predictor of mobility, usual activities, and pain/discomfort, whereas disease perception was significantly positively correlated with mobility and negatively correlated with pain/discomfort and anxiety/depression.

**Table 6:** Regression coefficients for predicting EQ-5D-5L health utility scores from Seattle Angina Questionnaire using indirect approach, *N* = 380

Variable	MO	SC	UA	PD	AD
SAQ PL	-12.6193^***^	-11.5837^***^	-10.2628^***^	-4.4646^***^	-0.8718
	[1.3646]	[2.0187]	[1.1946]	[1.0459]	[0.9153]
SAQ AS	-1.2609	-0.4644	-1.0368	-1.3777^*^	-0.7419
	[0.7910]	[1.7335]	[0.6768]	[0.5914]	[0.5756]
SAQ AF	-1.4301	-2.6338	-1.4646^*^	-1.6318^*^	-1.5527^*^
	[0.7593]	[1.4914]	[0.6879]	[0.6454]	[0.6109]
SAQ TS	-4.4563^*^	-2.3537	-3.8393^*^	-3.6361^*^	2.6952
	[1.7579]	[3.3780]	[1.6251]	[1.5482]	[1.4271]
SAQ DP	3.1240^*^	5.0511	0.2211	-5.2587^***^	-10.6382^***^
	[1.5290]	[2.7691]	[1.4063]	[1.3923]	[1.4331]
Cut1	-10.2116^***^	-4.5555^*^	-10.3147^***^	-10.3706^***^	-5.3762^***^
	[1.2489]	[1.8417]	[1.1832]	[1.1160]	[0.9003]
Cut2	-6.5813^***^	-1.9126	-6.5026^***^	-6.4055^***^	-3.4332^***^
	[1.1164]	[1.8522]	[1.0379]	[0.9733]	[0.8653]
Cut3	-3.3392^**^	-1.2472	-3.4244^**^	-2.6310^*^	1.7840
	[1.2704]	[1.9092]	[1.1673]	[1.1236]	[1.2772]

*Notes*: ^*^, *P* < 0.05; ^**^, *P* < 0.01; ^***^, *P* < 0.001;

MO, mobility; SC, self-care; US, usual activities; PD, pain/discomfort, AD, anxiety/depression

PL, physical limitation; AS, angina stability; AF, angina frequency; TS, treatment satisfaction; DP, disease perception. The coefficients were captured from ordered logit regression models in which Seattle Angina Questionnaire score multiplied by 100

## Discussion

In this study, we developed mapping algorithms from the SAQ to the EQ-5D-5L using both direct and indirect approaches. To the best of our knowledge, this is the first mapping algorithm based on the Chinese version of the EQ-5D for patients with CHD. According to the four performance indicators, mapping algorithms derived from the beta regression mixture model in the direct approach are recommended to estimate EQ-5D-5L values, and algorithms in the indirect approach have comparable or better predictive performance than the direct approach.

Compared with previous studies, the mapping models developed in this study have important differences. First, for the direct mapping approach, the beta regression mixture model outperformed the traditional OLS and Tobit regression models. The results of this study are similar to those of previous studies conducted on other diseases, which also found that the beta regression mixture model has lower MAE and RMSE than traditional regression models [30, 44]. As Gray explained, the “bespoke” beta regression mixture model is more appropriate for fitting the distribution of EQ-5D-5L, including inflation at boundary values, gap between full health, and next feasible value [32]. The MAE of the beta regression mixture model (0.0591) was much lower than that of Wijeysundera (0.088) based on OLS [17], and the RMSE (0.0864) was lower than that of Goldsmith (0.170) based on OLS [18]. Thus, the beta regression mixture model could decrease the bias of overestimating health utility values in poor health and underestimating values in good health, while traditional regression linear models typically lead to biased prediction of health utility. This could be because the mixture beta regression model is more robust and sensitive to fit pile-up values at boundaries and can capture multimodality of utility values [32]. Future mapping studies should apply the mixture beta regression model to other diseases.

Second, this study captures mapping algorithms in an indirect approach using an ordered logit model. Previous studies mapping the SAQ to EQ-5D did not implement an indirect approach [17–19]. Moreover, we found that the mapping algorithm in the indirect approach has a better predictive performance than that in the direct approach. The prediction errors of the indirect approach are greater than those of the indirect approach, while the correlation coefficients between observed and predicted health utility values captured from the indirect approach are higher than those from regression models in the direct approach. Thus, the indirect mapping algorithms reported in this study could not only facilitate the calculation of EQ-5D-5L scores using other Chinese measures but could also be generalized to predict health utility values using other country-specific measures.

Third, the coefficients of the five subscales of the SAQ have subtle differences from those in previous studies. As Wijeysundera and Goldsmith reported [17, 18], only physical limitation, disease perception, and angina frequency were significant predictors of EQ-5D in all regression models. This finding is consistent with those of the present study. However, we found that angina stability was significant in the beta regression mixture model. Furthermore, the results of the ordered logit model illustrate that angina stability is negatively significant with pain/discomfort, and treatment satisfaction is a significant predictor of mobility, usual activities, and pain/discomfort. As Wijeysundera explained, lack of conceptual overlap between SAQ and EQ-5D may lead to modest predictive ability of the mapping model [17]. Contrastingly, the results of indirect mapping in this study imply that there are variant overlaps between the five SAQ subscales and EQ-5D dimensions. Thus, to improve model predictive performance, an indirect approach should be implemented to map SAQ to EQ-5D in future research and practice.

This study had several limitations. First, all participants were recruited from a hospital in China using convenience sampling methods. The sampled data could not represent all patients with CHD in China. Future research should be conducted with a larger number of representative patients. Second, patients with serious comorbidities and hearing or vision impairments were excluded, which may have led to an overestimation of the mean health utility values. This could restrict the generality of the mapping algorithm. Moreover, patients with CHD and serious comorbidities were excluded in this survey. Thus, the mapping algorithm should be applied for patients with mild CHD. Thirdly, the mapping algorithms were validated using only an internal cross-validation method. External validation is desirable with an independent dataset to assess predictive performance. Lastly, this study included only 380 participants, and responses could not span all five levels of the EQ-5D questionnaire. This may decrease the performance of the ordered logit model using indirect approach.

## Conclusions

In conclusion, this study developed the first mapping algorithm to transform SAQ to EQ-5D among patients with CHD in China. This could promote the utilization of health economic evaluations in resource allocation policy-making. Furthermore, it provides an indirect mapping algorithm, which can be conveniently generalized to other countries or regions with country-specific measures.

## Electronic supplementary material

Below is the link to the electronic supplementary material.

Supplementary Material 1

Supplementary Material 2

## Data Availability

The data and code underlying this article will be shared on reasonable request to the corresponding author.

## List of abbreviations

CVD: Cardiovascular disease
CHD: Coronary heart disease
CUA: Cost-utility analysis
SAQ: Seattle Angina Questionnaire
SD: Standard deviation
MAE: Mean absolute error
RMSE: Root mean squared error
CCC Lin’s: concordance correlation coefficient
OLS: Ordinary least square regression
GLM: Generalized linear regression model
CLAD: Censored least absolute deviation
RMM: MM-robust regression
ALDVMM: Adjusted limited dependent variable mixture model
BM: Mixture beta regression model
