# Supplementary material for: Mapping the Seattle Angina Questionnaire to EQ-5D-5L in patients with coronary heart disease
Source: Health Qual Life Outcomes. 2023 Jul 3;21:64. doi: 10.1186/s12955-023-02151-9 (PMC10318834; doi:10.1186/s12955-023-02151-9)

# Supplementary Material 1: Data analysis results for baseline survey

## Table 1 Basic characteristics of participants in baseline and followed-up survey

| Variables | Baseline | Followed-up |
| --- | --- | --- |
| Total | 305 | 75 |
| Age (years), Mean (SD) | 62.90 (9.87) | 64.56 (9.06) |
| Gender |  |  |
| Male, *N* (%) | 157 (51.48) | 47 (62.67) |
| Female, *N* (%) | 148 (48.52) | 28 (37.33) |
| Disease type |  |  |
| Stable angina pectoris, *N* (%) | 78 (26.09) | 1 (1.45) |
| Unstable angina pectoris, *N* (%) | 199 (66.56) | 59 (85.51) |
| Myocardial infarction, *N* (%) | 22 (7.36) | 9 (13.04) |
| Duration of illness (years), Mean (SD) | 2.87 (5.42) | 2.36 (4.57) |
| EQ-5D-5L, Mean (SD) | 0.85 (0.14) | 0.98 (0.10) |
| Seattle angina questionnaire |  |  |
| Physical limitation, Mean (SD) | 68.82 (14.22) | 79.00 (12.21) |
| Anginal stability, Mean (SD) | 23.11 (22.46) | 89.33 (17.52) |
| Angina frequency, Mean (SD) | 56.95 (25.74) | 95.47 (10.17) |
| Treatment satisfaction, Mean (SD) | 65.17 (11.13) | 77.96 (7.05) |
| Disease perception, Mean (SD) | 48.36 (12.93) | 65.78 (6.06) |

## Table 2 Correlation coefficients of EQ-5D-5L and Seattle Angina Questionnaire subscales at baseline, *N*=305

|  | EQ-5D-5L | MO | SC | UA | PD | AD |
| --- | --- | --- | --- | --- | --- | --- |
| SAQ PL | 0.6353^**^ | -0.5907^**^ | -0.2560^**^ | -0.5627^**^ | -0.4939^**^ | -0.3349^**^ |
| SAQ AS | 0.5077^**^ | -0.3600^**^ | -0.1867^**^ | -0.3674^**^ | -0.4664^**^ | -0.3449^**^ |
| SAQ AF | 0.5337^**^ | -0.3946^**^ | -0.2434^**^ | -0.4079^**^ | -0.4386^**^ | -0.3629^**^ |
| SAQ TS | 0.5082^**^ | -0.3358^**^ | -0.1610^**^ | -0.3617^**^ | -0.4836^**^ | -0.3490^**^ |
| SAQ DP | 0.6042^**^ | -0.2723^**^ | -0.0865 | -0.3588^**^ | -0.5272^**^ | -0.5805^**^ |

^*^, *P*<0.05; ^**^, *P*<0.01.

## Table 3 Goodness of fit of direct and indirect mapping approach from baseline sample, *N*=305

| Model | Mean | Minimum | Maximum | MAE | RMSE | ρ | CCC |
| --- | --- | --- | --- | --- | --- | --- | --- |
| Observed | 0.8473 | 0.1690 | 1.0000 | - | - | - | - |
| OLS | 0.8473 | 0.5430 | 1.0827 | 0.0702 | 0.0930 | 0.7516 | 0.7219 |
| Tobit | 0.8637 | 0.5020 | 1.1483 | 0.0743 | 0.0970 | 0.7502 | 0.7406 |
| GLM | 0.8476 | 0.5954 | 1.1006 | 0.0723 | 0.0957 | 0.7340 | 0.6945 |
| CLAD | 0.8612 | 0.5957 | 1.0687 | 0.0694 | 0.0950 | 0.7508 | 0.6860 |
| RMM | 0.8721 | 0.6630 | 1.0742 | 0.0711 | 0.1042 | 0.7191 | 0.5901 |
| BM | 0.8484 | 0.3701 | 0.9992 | **0.0648** | 0.0885 | 0.7824 | 0.7565 |
| ALDVMM | 0.8517 | 0.5604 | 0.9971 | 0.0671 | 0.0925 | 0.7654 | 0.7088 |
| Indirect approach | 0.8399 | 0.3364 | 0.9933 | 0.0653 | **0.0869** | **0.7895** | **0.7737** |

*Notes*: Bold number indicate a best result on that indicator. OLS, order least square; GLM, generalized linear model; CLAD, censored least absolute deviations; RMM, MM-robust regression; BM, mixture beta regression model; ALDVMM, adjusted limited dependent variable mixture model. ρ, correlation coefficients between observed EQ-5D-5L scores and prediction; MAE, mean absolute error; RMSE, root mean square error, CCC, Lin’s Concordance correlation coefficient.

## Table 4 Predictive performance of direct and indirect mapping approach using cross validation method at baseline, N=305

| Model | MAE | RMSE | ρ | CCC |
| --- | --- | --- | --- | --- |
| OLS | 0.0716 | 0.0959 | 0.7330 | 0.7022 |
| Tobit | 0.0758 | 0.0997 | 0.7347 | **0.7406** |
| GLM | 0.0735 | 0.0981 | 0.7177 | 0.6779 |
| CLAD | 0.0743 | 0.1048 | 0.6822 | 0.5991 |
| RMM | 0.0726 | 0.1051 | 0.7100 | 0.5824 |
| BM | **0.0673** | **0.0938** | 0.7515 | 0.7230 |
| ALDVMM | 0.0687 | 0.0947 | 0.7481 | 0.6994 |

*Notes*: Bold number indicate a best result on that indicator. OLS, order least square; GLM, generalized linear model; CLAD, censored least absolute deviations; RMM, MM-robust regression; BM, mixture beta regression model; ALDVMM, adjusted limited dependent variable mixture model. ρ, correlation coefficients between observed EQ-5D-5L scores and prediction; MAE, mean absolute error; RMSE, root mean square error, CCC, Lin’s Concordance correlation coefficient.

## Table 5 Regression coefficients for predicting EQ-5D-5L health utility scores from Seattle Angina Questionnaire using direct approach, *N*=305

| Variable | OLS | TOBIT | GLM | CLAD | RMM | BM | | ALDVMM | |
| --- | --- | --- | --- | --- | --- | --- | --- | --- | --- |
|  |  |  |  |  |  | C1_mu | PM_ub | Com 1 | Com 2 |
| SAQ PL | 0.5140^***^ | 0.5691^***^ | 0.5766^***^ | 0.4157^***^ | 0.2231^***^ | 3.5299^***^ | 6.1925^**^ | 0.1049 | 0.8068^***^ |
|  | [0.0432] | [0.0496] | [0.0541] | [0.0354] | [0.0536] | [0.3704] | [1.9530] | [0.0548] | [0.0775] |
| SAQ AS | 0.0427 | 0.0447 | 0.0541 | 0.0426 | 0.0470 | 0.6528^*^ | 0.4106 | 0.0526 | 0.0485 |
|  | [0.0326] | [0.0377] | [0.0393] | [0.0236] | [0.0263] | [0.3016] | [1.0964] | [0.0367] | [0.0659] |
| SAQ AF | 0.0593^*^ | 0.0903^**^ | 0.0635 | -0.0025 | 0.0590^*^ | 0.6517^**^ | 1.4313 | 0.0377 | 0.1030 |
|  | [0.0283] | [0.0325] | [0.0347] | [0.0225] | [0.0280] | [0.2327] | [1.0820] | [0.0363] | [0.0561] |
| SAQ TS | 0.1312^*^ | 0.1456 | 0.1409 | 0.1745^**^ | 0.0604 | 0.5152 | 3.8092 | 0.0144 | 0.2081 |
|  | [0.0666] | [0.0758] | [0.0820] | [0.0539] | [0.0709] | [0.5782] | [2.7999] | [0.0766] | [0.1308] |
| SAQ DP | 0.1565^**^ | 0.2401^***^ | 0.1916^**^ | 0.1757^***^ | 0.2705^***^ | 1.5540^**^ | 10.2937^***^ | 0.3371^***^ | 0.0576 |
|  | [0.0580] | [0.0674] | [0.0713] | [0.0486] | [0.0588] | [0.4836] | [2.6934] | [0.0639] | [0.1117] |
| Constant | 0.2887^***^ | 0.1993^***^ | -0.8023^***^ | 0.3842^***^ | 0.5039^***^ | -1.4805^***^ | -15.3446^***^ | 0.6058^***^ | 0.0478 |
|  | [0.0387] | [0.0454] | [0.0486] | [0.0315] | [0.0485] | [0.3376] | [2.3705] | [0.0518] | [0.0761] |

*Notes:* ^*^, *P*<0.05; ^**^, *P*<0.01; ^***^, *P*<0.001; Com, component; C1_mu, Component 1 of mixture model; PM_ub, the inflation part of the model at perfect health.

OLS, order least square; GLM, generalized linear model; CLAD, censored least absolute deviations; RMM, MM-robust regression; BM, mixture beta regression model; ALDVMM, adjusted limited dependent variable mixture model. The coefficients were captured from each regression model in which Seattle Angina Questionnaire score multiplied by 100.

## Table 6 Regression coefficients for predicting EQ-5D-5L health utility scores from Seattle Angina Questionnaire using indirect approach, *N*=305

| Variable | MO | SC | UA | PD | AD |
| --- | --- | --- | --- | --- | --- |
| SAQ PL | -12.5101^***^ | -10.3171^***^ | -9.9352^***^ | -5.6518^***^ | -1.1552 |
|  | [1.4430] | [2.2262] | [1.2495] | [1.1571] | [0.9559] |
| SAQ AS | -0.7836 | -0.2071 | -0.3371 | -1.7568* | -0.1904 |
|  | [0.8956] | [1.8386] | [0.7954] | [0.7672] | [0.7160] |
| SAQ AF | -1.3752 | -2.8910 | -1.4708^*^ | -1.0511 | -1.5186^*^ |
|  | [0.7666] | [1.5113] | [0.6965] | [0.6667] | [0.6266] |
| SAQ TS | -4.2496^*^ | -2.7023 | -3.8455^*^ | -3.6611^*^ | 2.3859 |
|  | [1.7443] | [3.3251] | [1.6259] | [1.5828] | [1.4522] |
| SAQ DP | 3.3661^*^ | 4.6800 | 0.3133 | -4.4334^**^ | -10.3713^***^ |
|  | [1.5479] | [2.7755] | [1.4214] | [1.4236] | [1.4703] |
| Cut1 | -9.8277^***^ | -4.3267^*^ | -9.9636^***^ | -10.5812^***^ | -5.4992^***^ |
|  | [1.2483] | [1.8401] | [1.1887] | [1.1738] | [0.9270] |
| Cut2 | -6.2301^***^ | -1.5788 | -6.2121^***^ | -6.6147^***^ | -3.6173^***^ |
|  | [1.1186] | [1.8825] | [1.0467] | [1.0199] | [0.8911] |
| Cut3 | -2.4292 | -0.7510 | -2.5482 | -2.8113^*^ | 1.5810 |
|  | [1.4705] | [1.9979] | [1.3669] | [1.1548] | [1.2909] |

*Notes:* ^*^, *P*<0.05; ^**^, *P*<0.01; ^***^, *P*<0.001;

MO, mobility; SC, self-care; US, usual activities; PD, pain/discomfort, AD, anxiety/depression.

PL, physical limitation; AS, angina stability; AF, angina frequency; TS, treatment satisfaction; DP, disease perception. The coefficients were captured from ordered logit regression models in which Seattle Angina Questionnaire score multiplied by 100.

# Supplementary Material 2: Coefficients of regression models with covariates

## 1. OLS


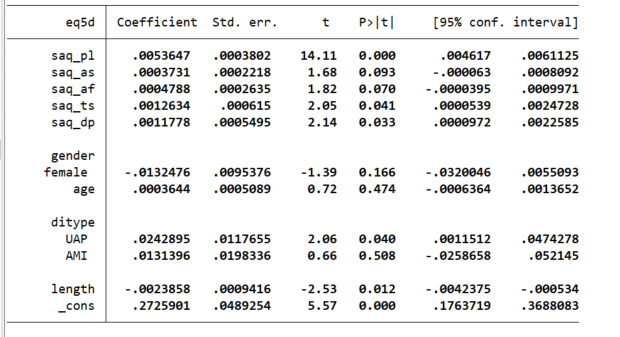


## 2. Tobit


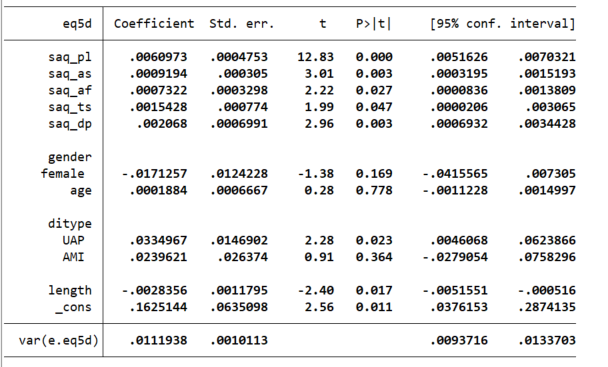


## 3. GLM


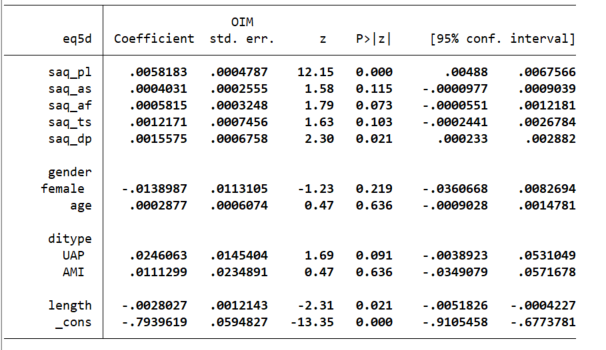


## 4.CLAD


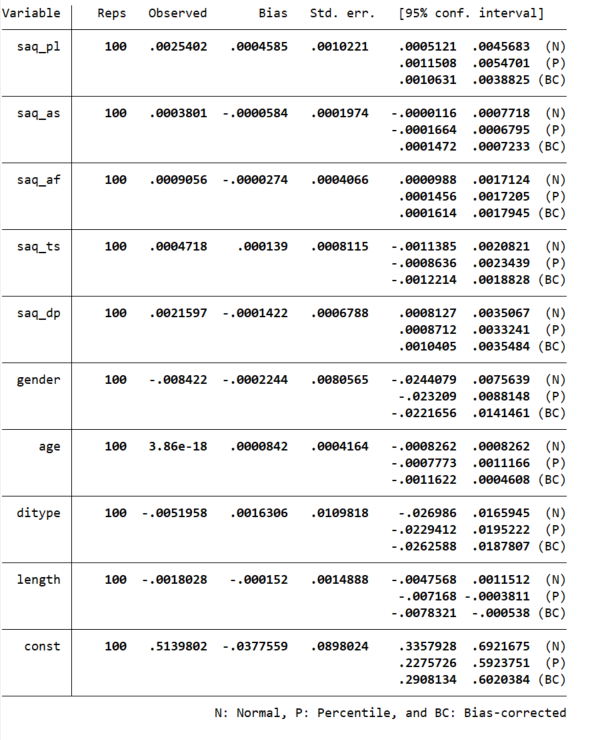


## 5. MM


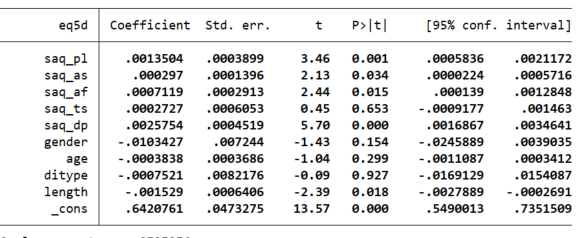


## 6. BM


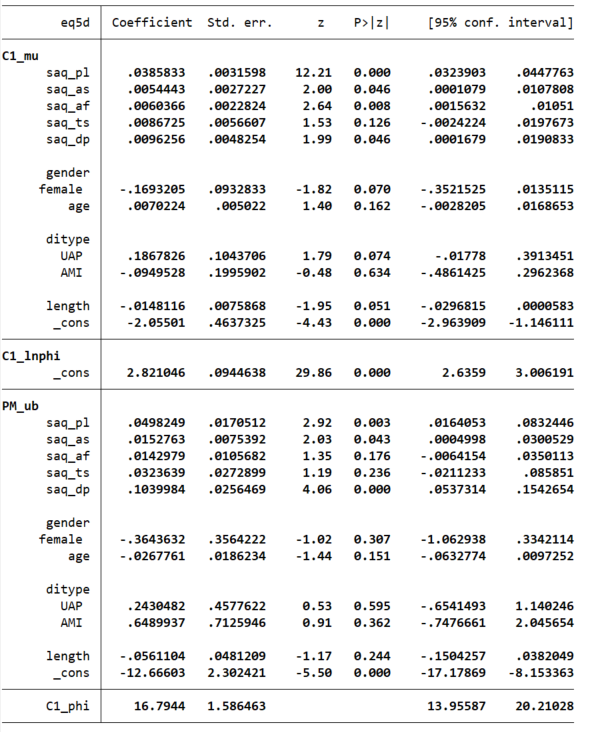


## 7. ALDVMM


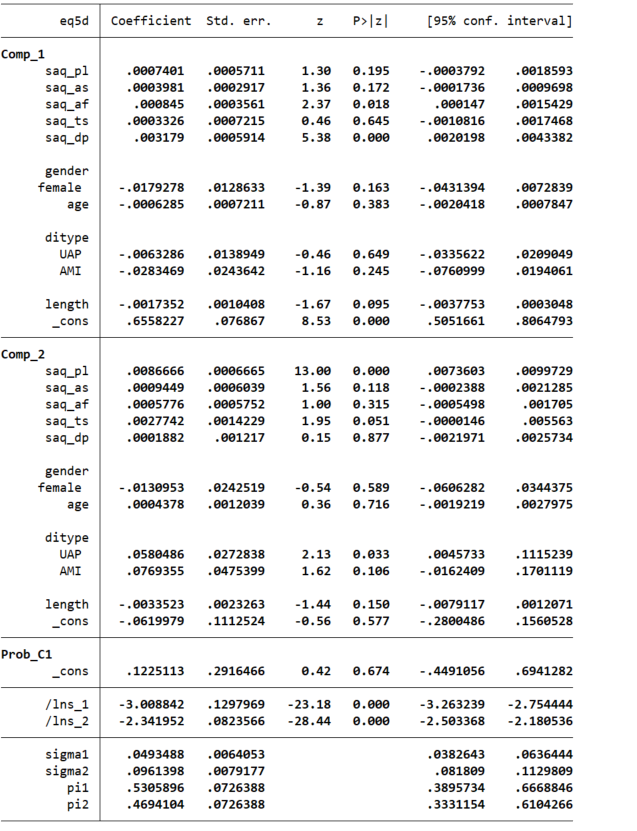


## 8. Response mappings

### 8.1 Mobility


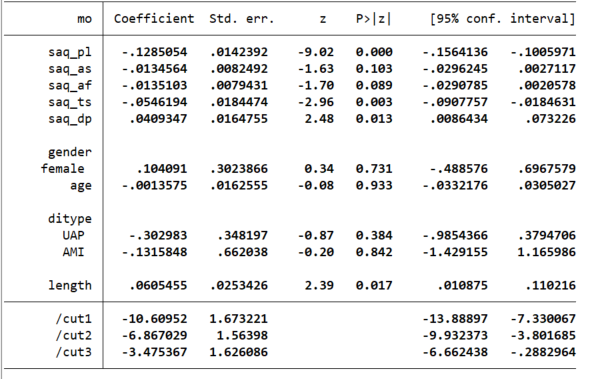


### 8.2 Self-care


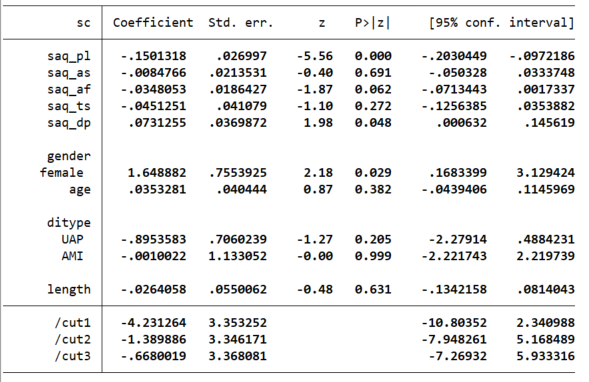


### 8.3 Usual activity


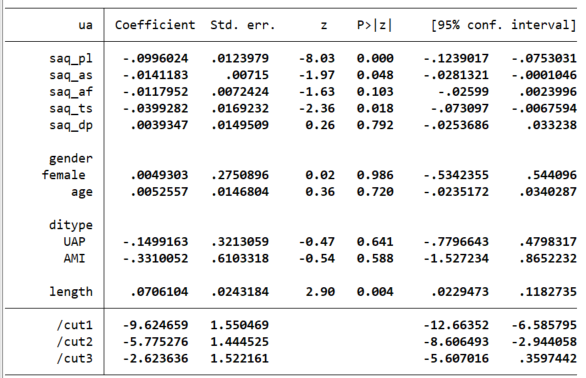


### 8.4 Pain/Discomfort


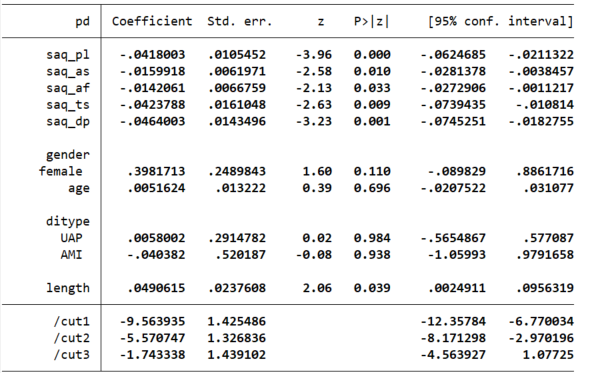


### 8.5 Anxiety


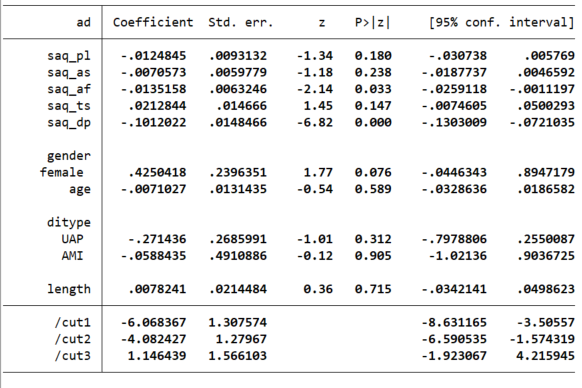


# Supplementary Material 3: Covariance matrix for direct and indirect mapping models

## 1.OLS


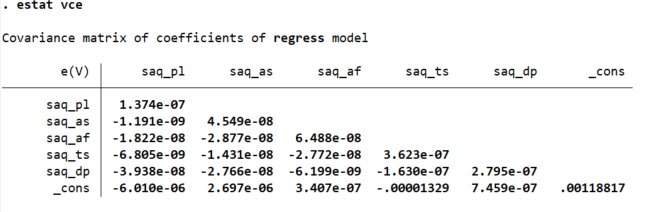


## 2. Tobit


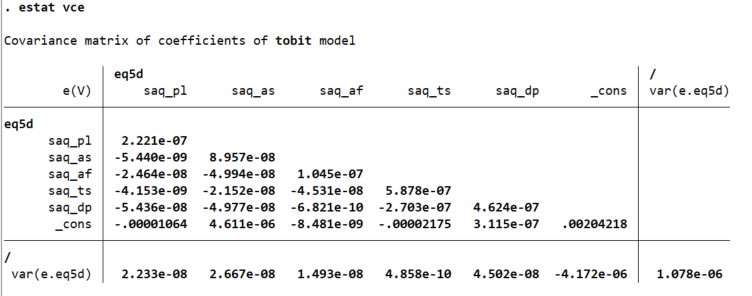


## 3. GLM


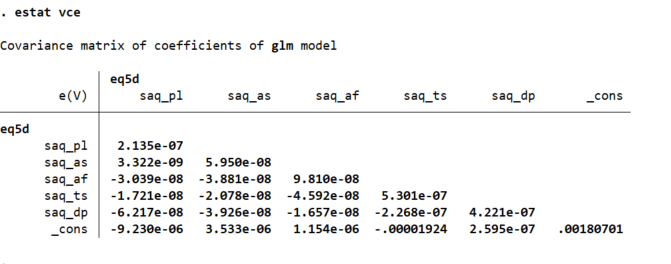


## 4. CLAD


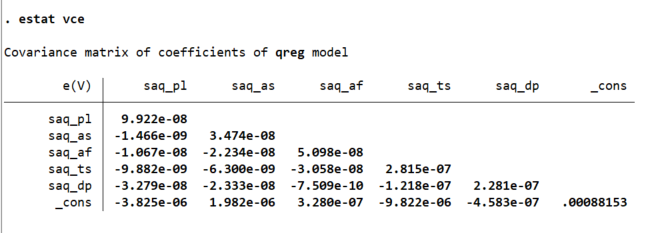


## 5. MM


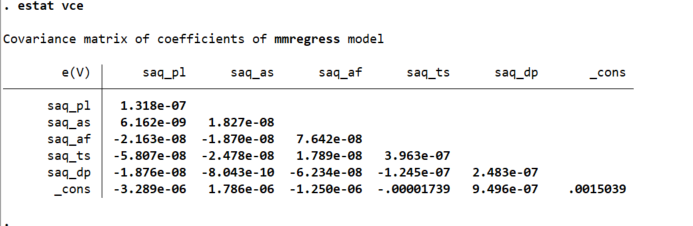


## 6. BM


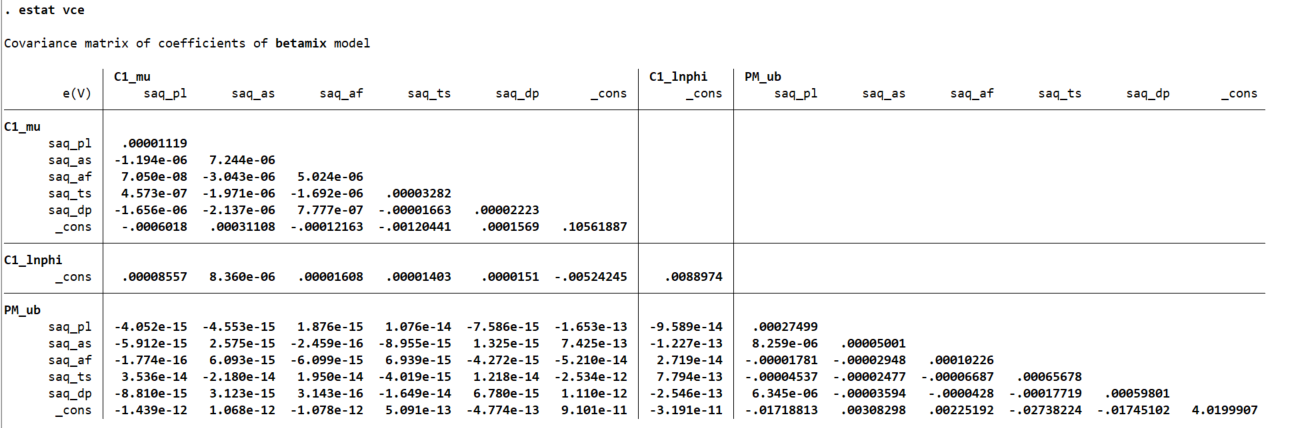


## 7. ALDVMM


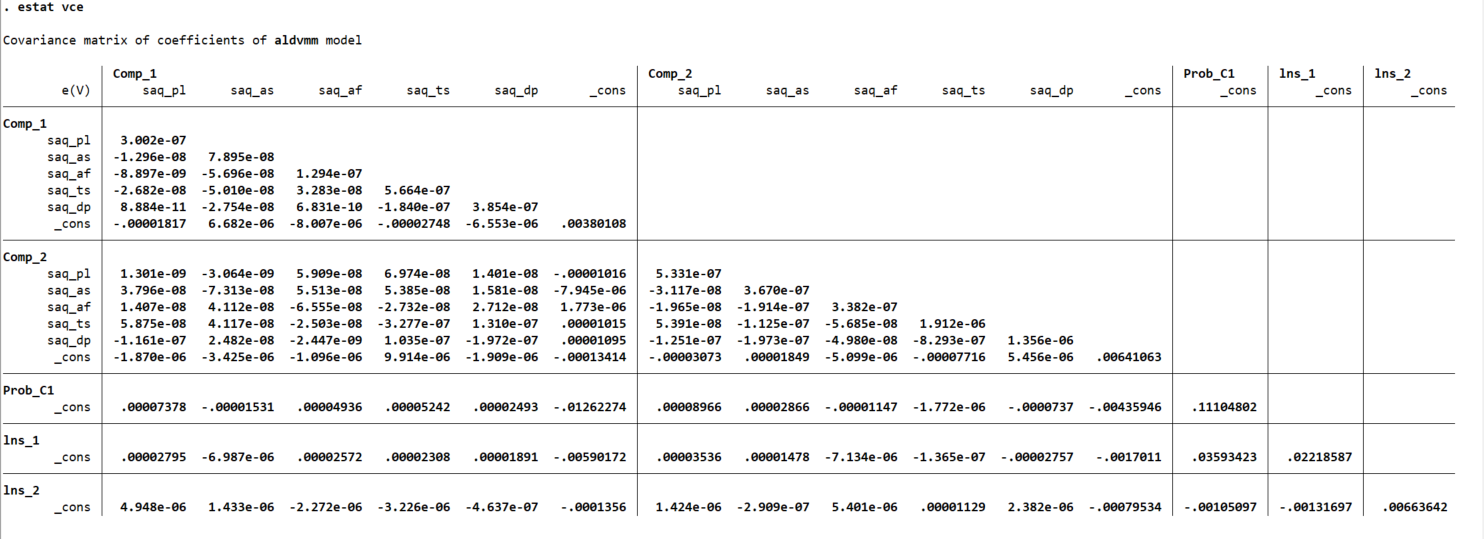


## 8. Response mappings

### 8.1 Mobility


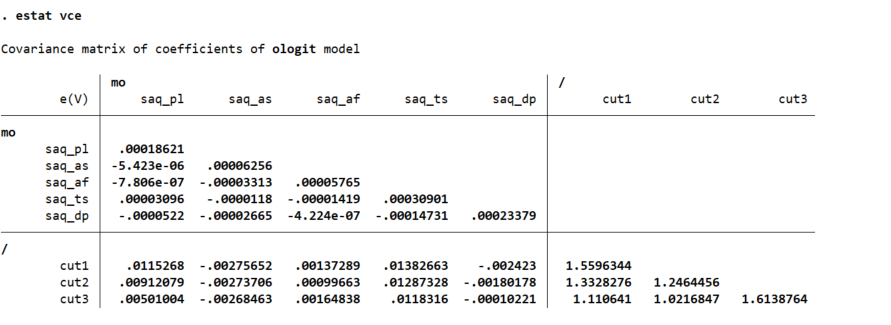


### 8.2 Self-care


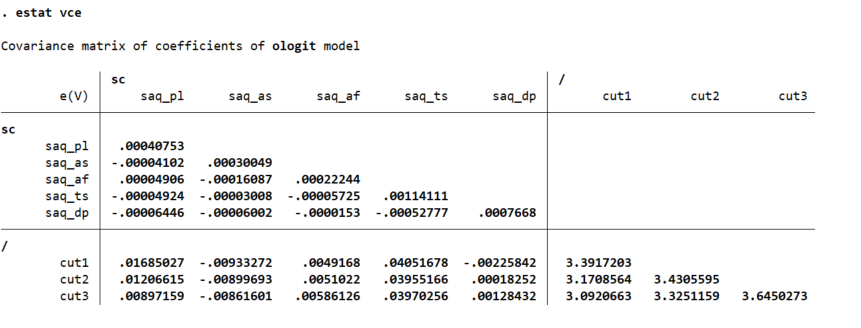


### 8.3 Usual activities


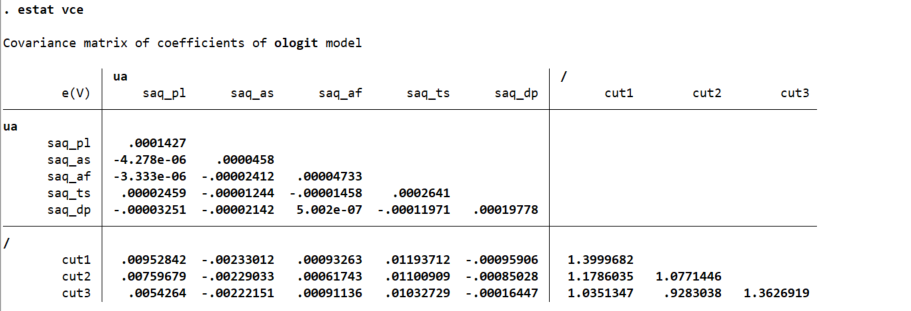


### 8.4 Pain/Discomfort


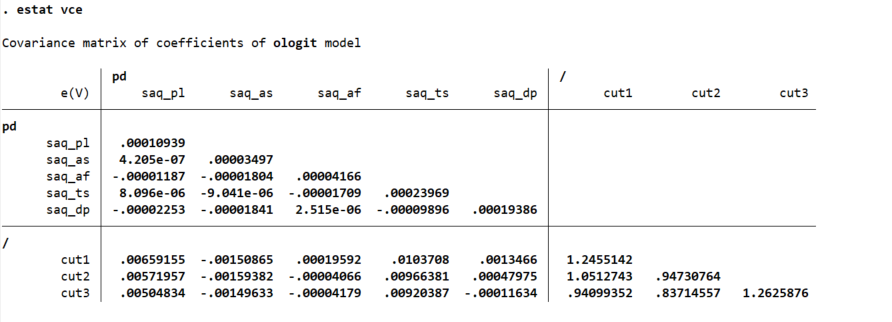


### 8.5 Anxiety/Depression


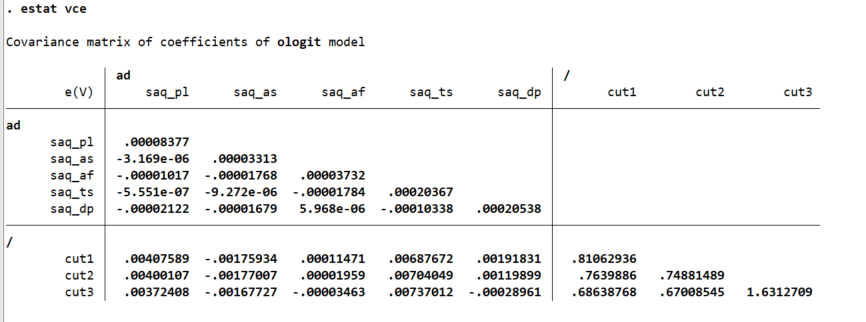

Supplement: Supplementary file 2 — Supplementary Material 2 [file 12955_2023_2151_MOESM2_ESM.docx]
